# Supplementary material for: Harnessing citizen science for marine conservation in Malta: a comparative analysis of GAM and MaxEnt models in bottlenose dolphin habitat mapping
Source: PeerJ. 2025 Aug 5;13:e19804. doi: 10.7717/peerj.19804 (PMC12333609; doi:10.7717/peerj.19804)
Supplement: Supplemental Information 1 [file peerj-13-19804-s001.docx]

**Harnessing Citizen Science for Marine Conservation in Malta: A Comparative Analysis of GAM and MaxEnt Models in Bottlenose Dolphin Habitat Mapping**

Francesca Soster^1†^, Tim Awbery^2†^, Nina Verité^3^, Timothy Zammit^3^, Kimberly Terribile^4^

^1^ Applied Research and Innovation Centre, Malta College of Arts Science & Technology (MCAST), Triq Kordin, Paola, PLA 9032, Malta

^2^ Scottish Association for Marine Science (SAMS), Oban PA37 1QA, United Kingdom

^3^ Discover the Blu, 76 Triq il-Lampuki, San Pawl il-Baħar SPB 3061, Malta.

^4^ Centre for Agriculture, Aquatics and Animal Sciences, Malta College of Arts, Science and Technology (MCAST), Luqa Road, Qormi, QRM 9075 Malta

^†^ These authors contributed equally to this work

**Supplementary Material 1**

**a) Visual Survey Protocol**

**Platform and Personnel**

Visual surveys were conducted from a small research vessel during daylight hours under favorable weather conditions (Beaufort ≤ 3). Two experienced observers were stationed at the bow, each scanning a 90° sector ahead of the vessel. The portside observer covered the arc from 270° to 360°, while the starboard observer monitored from 0° to 90°, relative to the vessel’s heading.

**Detection and Recording**

When a sighting occurred, the observers estimated the distance and angle to the sighted animals using a handheld compass and a calibrated rangefinder stick. Sightings were recorded in real-time on standardized datasheets. Environmental data (e.g., sea state, visibility, glare, swell, cloud cover) were recorded by a third researcher every 30 minutes or upon any change in environmental conditions. Information on other marine fauna and vessel presence was also noted.

**Trackline and Effort Data**

Survey effort was continuously logged using a handheld GPS device, with waypoints and tracklines saved for post-processing and spatial analysis.

**Permits**

All research activities were carried out under permit EP 0249/24, issued by the Environment and Resources Authority (ERA), authorizing marine mammal surveys in the waters surrounding the Maltese Islands.

**b) Citizen Science Reporting Protocol**

Members of the public were invited to contribute sightings of bottlenose dolphins via a standardized reporting form.

**Required Information:**

- **Date of sighting**
- **Time of sighting (hh:mm format)**
- **Location spotted (select or specify):**
  - Pembroke
  - St Paul’s Bay
  - Għadira Bay
  - Comino
  - Ċirkewwa Marine Park
  - Gozo (North East)
  - Gozo (South East)
- **GPS coordinates of sighting (Latitude and Longitude)**

**Species Identification Confidence:**

- Were you confident they were *bottlenose dolphins*? *(Identification aids were provided through an image guide.)*
  - Yes
  - No
  - Maybe

**Group Size and Composition:**

- How many dolphins did you observe?
- Were calves present? If yes, how many?

**Optional:**

- Upload a **photo** or **video** of the sighting if available.
